# Risk Predictors and Cognitive Outcomes of the Psychosocial Functioning of North American Older Adults During the COVID-19 Pandemic

**DOI:** 10.3390/healthcare13070792

**Published:** 2025-04-02

**Authors:** Kathryn Bolton, Lixia Yang

**Affiliations:** Department of Psychology, Toronto Metropolitan University, Toronto, ON M5B 2K3, Canada; kathryn.bolton@torontomu.ca

**Keywords:** COVID-19 pandemic, older adults, sociodemographic, psychosocial functions, cognition, coping

## Abstract

**Background**: The COVID-19 pandemic caused a global mental health deterioration. The disruption of older adults’ psychosocial functions is particularly concerning given their social support and technology use barriers. Despite a close relationship between social engagement and cognitive function in older adults, little is known about the cognitive consequences of older adults’ disrupted psychosocial functions in the context of the pandemic. **Aims**: This study aims to identify sociodemographic and COVID-19-related predictors for psychosocial functioning in North American older adults and to examine their associated cognitive outcomes. **Methods**: A sample of 95 older adults aged 60 and older (*M* = 68.85, *SD* = 6.458) completed an online study from January to July 2021, including a questionnaire on sociodemographic and COVID-19-related experiences, the Kessler-10 (K10) to assess psychological distress, Satisfaction with Life Scale (SWLS) and the UCLA Loneliness Scale Revised (UCLA) to index social function, and the Go/No-go Task (GNG) and Letter Comparison Task (LCT) as cognitive measures. **Results**: Higher psychosocial functioning was predicted by increased approach-based coping, being aged 65–69, 70–74, and over 75 years relative to being 60–64, and being in medium to excellent relative to poor health, while lower psychosocial functioning was predicted by increased avoidance based coping strategies and having average relative to low income. Psychosocial functioning was not seen to strongly predict cognitive functioning. However, being aged 75 years and older relative to being aged 60–64 predicted decreased accuracy on no-go trials and slower cognitive speed, and lower LCT accuracy was predicted by more avoidance-based coping and being in a religion other than Christianity or Catholicism (e.g., being spiritual). **Conclusions**: The results identified age, income, and health status as psychosocial function predictors among North American older adults, and increased age, religion, and use of avoidance-based coping strategies as predictors for decreased cognitive performance. The results shed light on future public health strategies to promote the psychosocial and cognitive health of older adults.

## 1. Introduction

As per the “psychological COVID-19 syndrome”, the pandemic caused a global mental health deterioration that negatively impacted psychosocial functions [1]. A wealth of research has been conducted to understand the impacts of COVID-19 on physical and mental health [2,3,4]. The impact may be particularly concerning for older adults. For example, older adults showed reduced mental health during the pandemic [3,5,6,7,8,9,10,11,12]. Social distancing during the pandemic increased the level of loneliness and social isolation, particularly among older adults [13,14,15]. Furthermore, heightened loneliness was associated with a higher risk of developing dementia symptoms [16]. However, it is unclear about the predictive factors for older adults’ psychosocial functioning and the cognitive outcomes of these functions in the context of such a prolonged stressor as the pandemic. The current study aims to fill these gaps.

### 1.1. The Psychosocial Impacts of the Pandemic

The pandemic and its related public health measures (e.g., lockdown and social distancing) had resulted in a global mental health deterioration, such as heightened symptoms of depression, anxiety, and stress, as well as increased psychological distress [17,18,19], and impacts were particularly concerning for Canadian population [1,19], and individuals with disabilities in America [20]. Given their higher COVID-19 virus contraction and related mortality rates [12,21,22], older adults also reported an increased risk of mental health deterioration, loneliness, lower life satisfaction, and cognitive decline during the pandemic [12,23]. Furthermore, older adults’ psychosocial functions are negatively impacted by reduced social support, low sense of community belonging, and barriers associated with using technology or the internet to maintain social connections during the pandemic [21,24].

Survey studies in Canada revealed a worsened psychosocial profile (e.g., declined life satisfaction) following the pandemic, particularly among immigrants from Asia [10]. However, the level of psychosocial functioning remained stable among healthy older adults over the course of the pandemic in Canada [25]. On the other hand, a large-scale longitudinal nationally representative survey in America revealed differentially lower loneliness and mental distress among older adults aged 65 and above, and a gradually reduced mental distress over time in the first year of the pandemic [26]. So, the results are a bit mixed even within North America, calling for more research on this population.

### 1.2. The Sociodemographic and COVID-Related Predictors

Past research has addressed psychological well-being and associated social, health, and demographic risk factors in the general population, specifically among older adults [5,6,7,8,26,27,28]. The results generally showed that being a woman, of a younger age (under the age of 40 years), with lower education, in lower financial/health status, being unpartnered, having more children, and being of a marginalized race or ethnicity all predict poorer mental health outcomes (such as increased psychological distress) during COVID-19 as compared to their counterparts [3,8,17,19,20,27,29,30,31,32,33,34,35]. For example, women were more likely to experience distress relative to men [17]; younger adults, those with lower education, financial satisfaction, or income, were more disrupted in mental health than older adults, those with higher education, better financial satisfaction, or income [12,27,28,36]. Research with older Chinese immigrants in Canada showed a protective effect of social support from friends for psychological wellbeing and life satisfaction [12]. The current study aims to systematically assess the prediction of a wide variety of sociodemographic variables and some COVID-related variables (e.g., symptoms, travel, contact, and compliance to protocol) for psychosocial functions among older adults in North America. The sample included approximately half from Canada (47%) and half from the United States (53%).

### 1.3. The Stress: Appraisal and Coping Theory

Chronic stress related to a prolonged pandemic may also lead to maladaptive coping and, consequently, poor psychosocial adaptation [37]. The *stress: appraisal and coping* theory describes stress as a dynamic relationship between the person and their environment in which cognitive appraisal shapes emotional responses and coping strategies [37,38]. Appraisal is a continuous process that determines an individual’s perceived success or likelihood to maintain well-being in the face of stressors [37,38]. Coping refers to cognitive and behavioural efforts to manage the impacts of stressors, and is influenced by the available psychological, social, environmental, and material resources [38]. Coping strategies include approach- (e.g., seeking information or attempting to solve a problem) and avoidance-based (e.g., active or passive disengagement with the stressor) [38,39]. Although the effectiveness of coping is largely context-specific, consistent avoidance-based coping is associated with negative mental health outcomes [37].

The COVID-19 pandemic can be seen as a “collective stressor” [29] that poses a threat far beyond the physical harm of the virus per se [5,18]. Public health preventative protocols (e.g., social distancing/isolation and lockdown) may increase separation from loved ones and disrupt one’s ability to perform daily routines/activities [2,6,40]. According to the stress: appraisal and coping theory, pre-existing stressors tied to social roles, socioeconomic status (SES), and health status can restrict cognitive and emotional resources required to effectively manage pandemic-related stressors (e.g., fear of getting sick, adjusting to a changed way of living, financial worry, uncertainty) and lead to poorer psychosocial functions in certain populations (e.g., women, marginalized population).

### 1.4. The Stress: Appraisal and Coping Theory in Relation to Cognition

Past research also suggests that executive functioning, particularly inhibitory control, may be related to successful coping. For example, avoidance-based coping was negatively correlated with inhibitory control and cognitive flexibility [41,42,43]. Moreover, those with higher inhibitory control exhibited differentially fewer depressive symptoms than those with lower inhibitory control, even when both groups used similar coping strategies [43]. On the other hand, it has been evident that adaptive coping is associated with better subjective well-being (i.e., life satisfaction), whereas maladaptive coping is associated with lower psychological well-being [37,44]. Taken together, past research revealed a reciprocal relationship between cognitive resources, successful coping, and psychosocial well-being. In light of the pandemic, compliance with COVID-19 protocols and adaptive coping strategies signal the necessity for adapting to pandemic-related challenges, particularly when the circumstances are misaligned with one’s values and beliefs—a central concept in stress: appraisal and coping theory.

Lower subjective well-being, as well as higher distress and loneliness, may be particularly concerning for older adults due to aging-related cognitive decline and the increased dementia and Alzheimer’s disease risk in late adulthood [16,45,46,47,48,49,50,51,52,53,54]. Considering the importance of social engagement in cognitive functions in older adults [55] and the association between heightened loneliness and a higher risk of developing dementia symptoms [16], it may be reasonable to assume that psychosocial functions could predict cognitive functions in older adults. The current study intends to address this specific question in light of the prolonged stress during the pandemic.

### 1.5. The Current Study

The current study addresses two research questions: (1) what sociodemographic and COVID-19-related variables predict psychosocial functioning in light of the pandemic? In light of the literature, it is hypothesized that certain sociodemographic factors (such as being a woman, lower education, lower financial satisfaction, lower health) and COVID-19-related variables (such as exposure to an infected person, experiencing COVID-19 symptoms, avoiding travel during COVID-19, and increased compliance to COVID-19 safety protocols) would predict higher levels of psychological distress and social functions during the pandemic. (2) Does psychosocial functioning predict cognitive performance in older adults? In light of the literature, it is hypothesized that better psychosocial functions will predict better cognitive performance, inhibition specifically, in older adults.

## 2. Materials and Methods

### 2.1. Participants

An a priori power analysis [56] determined that 73 participants needed to have a power of 0.90 to detect a small effect size (*f*^2^ = 0.13) in a multiple linear regression for one tested outcome measure (e.g., Psychosocial Index), at an alpha = 0.05. The final sample included 95 participants (women: *N* = 73) aged 60 through 88 years (*M* = 68.85, *SD* = 6.458). See Table 1 for sample characteristics.

Participants were recruited through MTurk (*n* = 50), from the Toronto Metropolitan Seniors Participant Pool (TMSPP; *n* = 43) and the local community (*n* = 10). Potential participants were self-screened based on the following inclusion criteria: (1) aged 60 and over; (2) proficient in English reading and writing; (3) have access to a computer with an internet connection; (4) without previous mental health disorders (e.g., mild cognitive impairment [MCI], generalized anxiety disorder, depression); (5) have not had a stroke or transient ischemic attack (TIA); (6) have not suffered a head injury involving losing consciousness for more than 10 min; (7) have not had chemotherapy; (8) have intact vision and hearing. Participants were also screened for dementia-related cognitive impairments with an online version of the Short Blessed Test (SBT) [57], with those who scored 6 or above being excluded. Additionally, participants who did not respond to at least half of the items for any of the outcome measures, who failed the attention check trials or those who entered a suspicious “bot” response (e.g., responded to “age” with an email) and those outliers who scored 2.5 standard deviations above or below the average mean on the cognitive tasks, were excluded from the final data analysis. A total of 103 older adult participants were recruited, with 18 removed from the final sample, six for an age of under 60, two for not reporting an age, as well as 8 for outlier performance on cognitive tasks.

### 2.2. Online Study Package

The online study was programmed on Gorilla.sc [58] including a survey and a cognitive task component. The survey collects sociodemographic and COVID-experience information and assesses psychosocial functions. The cognitive task component includes Go-No Go (GNG) and Letter Comparison Task (LCT) to assess executive functioning and processing speed. The data were collected from January to July 2021.

#### 2.2.1. Sociodemographic, Health Information, and ADL, and Coping Measure

Participants answered questions about their sociodemographic information (i.e., age, gender, residence country, marital status, educational attainment, English proficiency in listening, speaking, reading, and writing, employment status, perceived household income level, and religion,, health information), COVID-19 symptoms and experiences (i.e., COVID-19 symptoms, travel, COVID-19 contact, and compliance to protocol), and engagement of daily activities (indexed by the sum score of the Activities of Daily Living, ADL). The ADL was a novel scale including living skills outlined on various scales used to assess functional abilities in older adults [59,60,61].

Coping strategies were assessed using the Brief Approach/Avoidance Coping Questionnaire (BACQ), which captures approach (e.g., “I make an active effort to find a solution to my problems”, Items 1–6) versus avoidance (e.g., “I withdraw from other people when things get difficult”, Items 7–12) coping strategies utilized by individuals. It has shown satisfactory psychometric properties, with Cronbach’s α = 0.68 for all 12 items, and the concurrent validity with related COPE-Questionnaire [62] subscales at 0.34 and 0.37 for avoidance indices, and 0.50 to 0.57 for approach indices. A higher sum score in each subscale indicates utilizing more approach- or avoidance-based strategies

#### 2.2.2. Psychosocial Outcome Variables

The Kessler 10 (K10) is a valid test for non-specific psychological distress (i.e., anxiety and distress), with solid psychometric properties (Receiver Operating Characteristics [ROC] curve of 0.876 for disorders with Global Assessment of Functioning (GAF] scores of 0–70, and 0.955 for disorders with GAF scores of 0–50, high internal reliability, consistency of Cronbach’s α = 0.93) [63,64]. Higher sum scores index higher psychological distress.

The Satisfaction with Life Scale (SWLS) [65,66] captures the global evaluation of life satisfaction [62,63]. It has been validated for older adults [48]. The scale has a solid test-retest reliability (Cronbach’s α = 0.85) and a good convergent validity, with an individual and peer report correlation of *r* = 0.55, an individual and family report correlation of *r* = 0.57, and peer and family report correlation of *r* = 0.54 (all significant at *p* < 0.001) [67]. Higher sum scores indicate higher life satisfaction.

The UCLA Loneliness Scale Revised (UCLA) assesses perceived social isolation and loneliness. It is a valid test for emotional and social loneliness in older adults, with Cronbach’s α = 0.85 [68] and test-retest correlation of *r* = 0.73 [69]. Higher sum scores represent a lower level of loneliness.

The psychosocial measures were seen to be significantly correlated (K10 with SWLS: *r* = −0.470, *p* < 0.001; K10 with UCLA: *r* = 644, *p* < 0.001; SWLS with UCLA: *r* = 0.621, *p* < 0.001). For clarity purposes, we calculated a composite z-score of all the K10 (reverse coded so that increased scores indicate lower distress), SWLS, and UCLA to serve as a Psychosocial Index with higher scores indicating better psychosocial functions.

#### 2.2.3. Cognitive Outcome Variables

The GNG [70,71] measures inhibitory control by speed and accuracy in interference resolution (i.e., the ability to focus and respond to task-relevant information and filter out irrelevant information). In this task, participants were instructed to press the spacebar to a “go” stimulus (i.e., “A, B, C”), and withhold the pressing response to a “no-go” stimulus (i.e., “X”). The LCT [72] measures processing speed. In this task, participants were instructed to respond “same” by pressing J or “different” by pressing F to a pair of 3-, 6-, and 9-letter pair strings as quickly as possible. Composite scores will be calculated for the RT and accuracy across the 3-, 6-, and 9-letter pairs. Both cognitive tasks were modified to be delivered online and capture the optimal performance of older adults in a computerized platform [70,73]. We chose inhibitory control and processing speed as they have been shown to be related to coping and depressive symptoms, as well as age-related cognitive decline [42,43,74,75,76,77].

### 2.3. Data Analysis

The data analysis was performed in IBM SPSS 28, with identifying information (i.e., IP addresses) removed before the analysis. Frequencies were run on categorical sociodemographic variables, and these variables were then recoded by merging levels with very small sample sizes into meaningful 3-, 4-, and 5-level categories. Missing values were replaced with an average for each measure and each participant. Regarding health status, this was captured on a 10-point scale and categorized into three distinct groups (1–5: poor to medium; 6–8: medium to good, 9–10: excellent) determined by inspection of the score distribution in the current sample, with the aim to create logical distinctions while maintaining relative balances across the categories. This was done in adherence to common methodological practice and has been shown to produce meaningful comparisons between categorical groups [78].

Multiple linear regression analyses were performed separately for each psychosocial and cognitive function outcome index. The regression models included both categorical variables (e.g., dummy-coded age groups, with 59–64 as reference) and continuous predictors (e.g., BACQ, ADL).

For the first research question, the predictor variables were sociodemographic, COVID-related variables, ADL, and BACQ score, and the outcome variables were the psychosocial functions, represented by the Psychosocial Index. Univariate analysis of variance (ANOVA) models for categorical variables and Pearson correlations for continuous variables were employed to identify potentially significant sociodemographic (i.e., sociodemographic, health, and ADL) for the Psychosocial Index. Based on the convention [79], those with a *p* < 0.20 in the ANOVA and Pearson correlations were identified as potential predictors. This was done to create parsimonious models to reduce the likelihood of overfitting. All identified potential predictors were entered into the subsequent multiple linear regression models, one for the ¨Psychosocial Index” outcome variable.

For the second research question, the primary predictors were the psychosocial factors, covariates were sociodemographic, COVID-related variables, and ADL, and the outcome variables were four cognitive performance variables: the go- and no-go trial accuracy, LCT accuracy, and Cognitive Speed Index (i.e., a composite score of the z scores of GNG go-trial RT and LCT RT). The same steps were followed to identify potential sociodemographic and COVID-19-related predictors for each of the four cognitive outcome measures. The Psychosocial Index, coping (i.e., BACQ), and identified covariates were entered into the multiple linear regression models, one for each cognitive outcome variable. The choice to include the Psychosocial Index, coping, and ADL as predictors in the models, regardless of their correlations with each cognitive outcome score, was driven by our research interest and hypothesis that cognitive performance would be predicted by psychosocial functions, which is related to coping strategies, as well as cognition’s relation to functional abilities in older adulthood [42,80].

### 2.4. Ethical Considerations

This study was approved by the Toronto Metropolitan’s Research Ethics Board (2020-207-1). All participants were informed of the research objectives, assured of confidentiality, and their right to discontinue participation without consequence.

## 3. Results

### 3.1. Sociodemographic and COVID-Related Predictors for Psychosocial Functions

Table 1 reports the sample characteristics, along with psychosocial outcomes stratified by categorical sociodemographic and COVID-related variables. Table 2 reports the Pearson correlations between continuous predictive variables (i.e., ADL, BACQ) and psychosocial outcomes. Both ADL and BACQ showed significant correlations and were thus identified as potential predictors to be entered into the subsequent multiple linear regression models (Table 3).

Based on the ANOVA results, the following sociodemographic and COVID-related variables were identified as potential predictors (*p* < 0.20): age, gender, income, health status, COVID symptoms, and COVID contact. These identified predictors will be entered into the subsequent multiple linear regression model (Table 3).

Table 3 shows the multiple linear regression results for psychosocial functions. The regression model can explain a significant 50% of the variance (*R^2^* = 0.495) in the Psychosocial Index, *F* = 6.414, *p* < 0.001. The following predictors were identified for increased psychosocial function: more approach based coping (*β* = 0.200, *p* = 0.025, 95% CI [0.007, 0.103]), being aged 65–69 (*β* = 0.240, *p* = 0.013, 95% CI [0.104, 0.867]), 70–74 (*β* = 0.223, *p* = 0.016, 95% CI [0.088, 0.846]), and 75 and older (*β* = 0.200, *p* = 0.025, 95% CI [0.007, 0.103]) relative to being 60–64 and being in medium to good (*β* = 0.414, *p* = 0.006, 95% CI [0.215, 1.224]) or excellent health (*β* = 0.509, *p* = 0.001, 95% CI [0.372, 1.488]) relative to being in poor to medium health. The following predictors were identified for decreased psychosocial function: more avoidance-based coping (*β* = −0.298, *p* < 0.001, 95% CI [−0.111, −0.030]), having average relative to lower perceived income (*β* = −0.252, *p* = 0.017, 95% CI [−0.785, −0.078]).

### 3.2. Psychosocial and Sociodemographic/COVID Prediction for Cognition

Table 1 reports cognitive outcomes stratified by categorical sociodemographic and COVID-related variables. Based on the ANOVA (Table 1) and Pearson correlation results (Table 2), the following categorical covariates were identified (*p* < 0.20): five for No-Go Accuracy (age, COVID-19 contact, COVID-19 travel, ADL, and BACQ avoid), three for Go Accuracy (gender, education, and BACQ avoid), two for LCT Accuracy (age, and religion), and one for Cognitive Speed Index (age). These identified predictors will be entered in the subsequent multiple linear regression models (Table 4).

Given our primary interest in the prediction of psychosocial functions in cognitive outcome, the Psychosocial Index, continuous predictors, as well as the ADL, and BACQ continuous covariates, were entered in the multiple linear regression models on four cognitive outcome variables (one for each), along with the sociodemographic and COVID-related covariates identified in the ANOVA models. Table 4 shows the regression results for cognitive outcomes.

The regression on No-Go Accuracy was significant (*F* = 3.319; *R*^2^ = 0.249; *p* = 0.002). Lower accuracy on No-Go trials was predicted by being aged 75 and above relative to aged 60–64 (*β* = −0.445, *p* < 0.001, 95% CI [−0.211, −0.075]). All the other predictors were not significant (*p*s > 0.052). The regression on LCT accuracy was significant (*F* = 2.028; *R*^2^ = 0.169; *p* = 0.045). Lower accuracy on LCT was predicted by more avoidance coping (*β* = −0.228, *p* =.047, 95% CI [−0.009, 0.000]) and being of “other” religion relative to Christian/Catholic (*β* = −0.231, *p* = 0.039, 95% CI [−0.081, −0.002]). No other psychosocial or covariate was significant (*p*s > 0.111). The regression on the Cognitive Speed Index was significant (*F* = 0.016; *R*^2^ = 0.166; *p* = 0.016), with longer response times by those aged 75 years and over than those aged 60–64 (*β* = 0.435, *p* < 0.001, 95% CI [0.484, 1.402]). All the other predictors were not significant (*p*s > 0.091). The regression Go Accuracy model was not significant (*p* = 0.206).

## 4. Discussion

This study addresses two research questions. First, what sociodemographic and COVID-19-related variables predict older adults’ psychosocial function? Second, does the psychosocial functioning of older adults predict their cognitive performance? The discussion below was organized to address these two questions.

### 4.1. Sociodemographic and COVID-19-Related Predictors for Psychosocial Functions

For the first research question, these results identified some important sociodemographic predictors for psychosocial functioning among older adults during the pandemic. Specifically, better psychosocial functioning was predicted by increased reliance on approached-based coping strategies, being aged 65–69, 70–74, and over 75 (as compared to being 60–64), and being medium to good or excellent health (as compared to being in poor health), with a low income (relative to average income).

Previous research has shown that older adults exhibit resiliency in light of the threat of COVID-19, which then results in relatively more positive mental health outcomes relative to younger adults [8,17,26,49,81]. Consistently, the current study showed better psychosocial wellbeing for older-old (i.e., 65 or above) than young-old (i.e., 60–64) adults. This may reflect a buffering effect of growing resilience with aging (e.g., [26]). While the current sample used both approaches, the score was significantly higher for approach-based than avoidance-based coping strategies (*t* (94) = 14.509, *p* < 0.001), which further protects older adults’ psychosocial functions. The positive psychosocial benefits of approach-based coping relative to avoidance-based coping provide solid support for the *stress: appraisal and coping* theory [37]. Potentially, having experienced more stressful situations across their lifespan has given older adults the ability to develop more adaptive coping strategies which may further contribute to their psychosocial wellbeing despite the prolonged pandemic-related stress.

In the literature, factors associated with higher SES (e.g., higher perceived income, owned home) tend to be associated with better mental health outcomes, such as increased life satisfaction [19,71,72,82]. This may be a result of increased access to health-promoting resources (e.g., being able to afford healthcare and access to nutritious food) and decreased stress associated with financial or housing insecurity. Therefore, those with higher SES may have less stress related to the worry about financial or housing insecurity alongside the other stressors during the pandemic. However, there was a counterintuitive finding in the current study showing that having an average income as compared to low predicted worse social functioning. This finding may represent unique financial pressures experienced by individuals in average-income households during the early years of the COVID-19 pandemic. Research has shown that individuals in middle-income brackets during COVID-19 may have experienced financial stress as a result of decreased working hours leading to decreased income, without the support that is usually granted to lower-income individuals [83]. This could contribute to a heightened perception of financial risk, which has been seen to predict increased depressive and anxiety symptoms [84]. This may help explain why those in the average, compared to lower, income group had significantly lower psychosocial functions.

Furthermore, health status was a commonly reported predictor of psychosocial functioning, which has been demonstrated in previous literature on distress, life satisfaction, and loneliness [20,29,32,85,86]. This might have unique implications in light of the pandemic, as poor health increases the vulnerability to the COVID-19 virus infection, thus exacerbating health anxiety [29] and increased social isolation. Additionally, their loved ones may have been more likely to adhere to social distancing and isolation policies more strictly due to older adults being at an increased risk of COVID-19, and this may have reduced the availability of formal and informal caregiving, resulting in diminished well-being.

### 4.2. Psychosocial and Sociodemographic Predictors for Cognitive Performance

For the second research question, the results show that psychosocial functions do not strongly predict cognitive functions in the current sample. However, decreased accuracy on no-go trials and having slower cognitive speed were significantly predicted by being aged 75 years and older relative to being 60–64, while decreased LCT accuracy was predicted by increased reliance on avoidance-based coping strategies and being from “other” religion (such as being spiritual, a humanist, protestant, or a Taoist) relative to being of a Christian or Catholic faith. Therefore, our hypothesis was not well supported by the current sample. Nevertheless, avoidance-based coping, being related to poorer psychosocial functioning, predicts poorer cognitive accuracy in a speed task.

We speculate that the lack of a direct prediction of psychosocial function for cognitive performance may be due to a few reasons. First, the sample characteristics may have reduced the likelihood that we would detect an effect. The current sample reported higher psychosocial functions across the psychosocial measures, with most participants’ scores indicating they are “likely to be well” on the K10, “slightly satisfied” to “extremely satisfied” with their life on the SWLS, and are experiencing a low degree of loneliness on the UCLA. Therefore, there could have been little room for variability in psychosocial functions to detect its impacts on cognitive performance. Furthermore, the cognitive tasks may be not sufficiently challenging, as reflected in their high accuracy rate (many at 90%) across the GNG and LCT tasks. The restricted variability in both psychosocial and cognitive performance might have restricted our ability to detect this prediction. A future study may further examine this relationship in clinical populations or those with larger variability in psychosocial and cognitive functioning to better understand the relationship between these variables.

Secondly, we only captured their functioning at one time point, and thus, were unable to capture the potential emergence of the relationship between psychosocial and cognitive functioning over time. It may be that psychosocial factors have a cumulative effect on cognitive functions, such that prolonged distress leads to diminished cognitive abilities over time. This might be particularly relevant for those who engage in more avoidance-based coping strategies. Considering that increased avoidance-based coping was associated with lower LCT accuracy, there might be a potential relationship between coping and processing speed. This may reflect an accuracy-speed trade-off to prioritize speed over accuracy associated with avoidance coping. Given that avoidance-based coping predicts poorer psychosocial functions, we speculate that when the tasks are sufficiently resource-demanding, we might detect this relationship, with poorer psychological functions that may deplete overall resources and make it harder to compensate for cognitive deficits naturally occurring with the biological aging process.

Moreover, psychosocial functioning may change over time. Although one may be able to maintain their psychological well-being in the short term, their resources to effectively cope and maintain their well-being may diminish when there is no reprieve from the stressor. Hence, future studies may examine this relationship in a longitudinal design to examine the changes in psychosocial functions over the course of a prolonged stressor event (e.g., a natural disaster) and the cumulative effect on cognitive performance.

Although not the primary focus of the second research question, it is worth discussing some covariates that were significant predictors of cognitive functions. Specifically, an age effect was detected, with those aged 75 years and older (compared to those aged 60–64) showing decreased accuracy on no-go trials and longer reaction times on the Cognitive Speed Index, showing a well-established age-related decline in processing speed, presumably due to the biological aging process [77,87,88]. In light of the current study, this decreased efficiency in processing speed may be related to their choice of avoidance-based coping (as seen with LCT accuracy). Future studies are needed to replicate and extend the findings through a longitudinal design to capture emerging trends of these relationships.

As the current study utilized MTurk to recruit part of the sample, we also analyzed the data to determine that there were performance differences across recruitment conditions, as has been seen in previous research [89,90,91]. The univariate ANOVAs were conducted to compare participants recruited through MTurk with those from other sources on cognitive outcomes. MTurk participants demonstrated better interference resolution (GNG no-go accuracy: MTurk: *M* = 0.936, *SD* = 0.130, Other sources: *M* = 0.879, *SD* = 0.128), *F* (1, 93) = 4.609, *p* = 0.034), and faster processing speed (LCT RT: MTurk: *M* = 2499.594, *SD* = 654.838, Other sources: *M* = 2836.106, *SD* = 698.139), *F* (1, 92) = 5.782, *p* = 0.018) compared to those recruited from other sources. This may reflect practice effects due to familiarity with computerized tasks. However, the main reported results on cognitive outcomes remained the same after controlling for recruitment source (included as a covariate; *p*s ≥ 0.170).

### 4.3. Limitations

The current study has a number of limitations. The nature of the online study contributed to a sampling bias, potentially evidenced by the prevalence of well-educated individuals with technology efficacy and high scores on cognitive tasks. This may restrict the generalizability of the results to those who do not have access to a computer with the appropriate accessories (i.e., keyboard, speakers, internet) and those who do not have technology competency and efficacy. These factors may have

Another factor to consider is that the online study was completed in the participants’ homes, with virtually no control over how or by whom it was completed. Participants may not have been able to secure an environment free from distractions while completing the study, which may have affected their responses to the measures. There is also a possibility that those who participated were not honest with the information they provided. Completion of the online study required intact sensory perception and fine motor skills (such as typing on a keyboard), which may have excluded those older adults with vision, hearing, or fine motor skill impairments.

As data was collected at a single time point, the cumulative effects of decreased psychosocial functions on cognition cannot be determined. The sample reported having relatively positive psychosocial functioning and were proficient in the cognitive tasks. Therefore, future studies should focus on collecting data at multiple timepoints, and explore a more varied sample of individuals who may be experiencing increased distress, loneliness, and reduced life satisfaction.

Racial and ethnic identity was not recorded in this study. Therefore, it is impossible to explore how these identities relate to the psychosocial and cognitive functioning of older adults during the COVID-19 pandemic. Finally, the associations found in this study are purely correlational, therefore, no causation inferences can be drawn.

## 5. Conclusions

Despite these limitations, the current study provides valuable information on how older adults in North America have experienced the COVID-19 pandemic. It has identified some important sociodemographic (i.e., age, income, and health status) risk factors for older adults’ psychosocial functioning. Furthermore, it explored the prediction of psychosocial functioning for cognitive performance, along with sociodemographic and COVID-related predictors. The results suggested that age and avoidance-based coping may be important factors to consider for inhibitory control and processing speed. The findings may inform interventions that target psychosocial functions and encourage approach-based coping strategies to promote the psychosocial and cognitive well-being of older adults.

## Figures and Tables

**Table 1:** Sample characteristics and ANOVA results on psychosocial function and cognitive outcomes, strategies by categorical variables.

Variables		Psychosocial Index		GNG No-Go Accuracy		GNG Go Accuracy		LCT Accuracy		Cognitive Speed Index	
	N (%)	*M*	*F (p)*	*M*	*F (p)*	*M*	*F (p)*	*M*	*F (p)*	*M*	*F (p)*
Gender			**1.871**		0.578		**4.447**		0.084		1.148
Woman	79 (77)	0.275	**(0.175)**	0.871	(0.449)	0.957	**(0.038)**	0.969	(0.772)	0.010	(0.287)
Man	23 (22)	0.005		0.846		0.936		0.964		0.253	
Age			**1.928**		**3.707**		1.127		**2.593**		**1.846**
60–64	37 (36)	−0.252	**(0.132)**	0.896	**(0.015)**	0.959	(0.343)	0.955	**(0.059)**	−0.089	**(0.146)**
65–69	23 (22)	0.225		0.866		0.947		0.979		0.037	
70–74	21 (20)	0.290		0.900		0.946		0.999		0.006	
75 and over	20 (19)	0.295		0.772		0.934		0.933		0.573	
Marital status			1.232		1.091		0.990		0.795		0.660
Partnered/Married	51 (50)	0.148	(0.297)	0.847	(0.341)	0.953	(0.376)	0.963	(0.455)	0.094	(0.520)
Divorced	21 (20)	0.321		0.892		0.946		0.954		0.003	
Widowed/single	31 (30)	−0.050		0.837		0.939		0.982		0.298	
Education			1.528		1.060		**3.878**		0.684		0.571
High school or lower	15 (15)	0.219	(0.223)	0.891	(0.351)	0.933	**(0.025)**	0.965	(0.508)	0.083	(0.567)
Post-secondary	56 (54)	−0.056		0.858		0.941		0.955		0.033	
Graduate school	32 (31)	0.256		0.827		0.965		0.979		0.278	
Employment			0.010		0.439		0.000		0.079		0.792
Retired/Unemployed	67 (65)	0.149	(0.921)	0.869	(0.510)	0.946	(0.998)	0.969	(0.779)	0.230	(0.376)
Employed	36 (35)	0.130		0.848		0.946		0.964		0.033	
Income			**6.633**		0.500		0.269		1.398		0.113
Low	29 (28)	0.304	**(0.002)**	0.857	(0.609)	0.947	(0.765)	0.976	(0.253)	0.130	(0.893)
Average	48 (47)	−0.273		0.876		0.943		0.947		0.076	
High	25 (24)	0.388		0.843		0.950		0.976		0.189	
Religion			1.208		1.483		0.188		**2.452**		0.382
Christian/Catholicism	56 (54)	0.098	(0.304)	0.873	(0.233)	0.949	(0.829)	0.980	**(0.093)**	0.124	(0.684)
None	28 (27)	0.345		0.885		0.947		0.985		0.009	
Other	19 (18)	−0.024		0.818		0.943		0.935		0.262	
Health status			**9.298**		1.450		0.535		0.020		0.360
Poor to medium	9 (9)	−0.657	**(<0.001)**	0.806	(0.241)	0.937	(0.588)	0.963	(0.981)	0.133	(0.698)
Medium to good	59 (57)	0.373		0.883		0.950		0.968		0.218	
Excellent	35 (34)	0.703		0.887		0.952		0.969		0.044	
COVID-19 symptom			**1.957**		0.151		0.491		0.356		0.006
No symptoms	68 (66)	0.265	**(0.166)**	0.864	(0.698)	0.943	(0.486)	0.972	(0.553)	0.105	(0.798)
At least one symptom	35 (34)	0.014		0.853		0.949		0.961		0.159	
COVID-19 contact			**5.205**		**3.175**		0.028		0.514		0.774
No contact	97 (94)	−0.432	**(0.025)**	0.785	**(0.079)**	0.948	(0.868)	0.949	(0.475)	0.384	(0.382)
Yes contact	5 (5)	0.712		0.932		0.944		0.984		−0.121	
COVID-19 travel			0.831		**3.518**		0.000		0.499		0.751
No (within 14 days)	98 (95)	0.392	(0.365)	0.944	**(0.064)**	0.946	(0.993)	0.947	(0.482)	−0.143	(0.389)
Yes (within 14 days)	4 (4)	−0.112		0.773		0.946		0.986		0.406	
Compliance to COVID-19 protocol			0.043		0.079		0.002		0.002		1.194
Low to Moderate	49 (48)	0.123	(0.836)	0.862	(0.780)	0.946	(0.963)	0.967	(0.967)	0.234	(0.278)
High	54 (53)	0.157		0.855		0.947		0.966		0.029	

Note. M = Mean, SD = Standard Deviation. All parenthesized frequency percentages have been rounded to the nearest whole number. Bold *F (p)* values refer to the variables identified as potential predictors for the corresponding outcome measures (*p* < 0.20).

**Table 2:** Pearson correlations between continuous predictors and psychosocial and cognitive outcome variables.

Variables		Psychosocial Index		GNG No-Go Accuracy		GNG Go Accuracy		LCT Accuracy		Cognitive Speed Index	
	*M (SD)*	*r*	*p*	*r*	*p*	*r*	*p*	*r*	*p*	*r*	*p*
ADL	16.12 (2.464)	0.274	0.005	0.141	0.155	−0.089	0.369	−0.147	0.141	−0.059	0.559
BACQ: Approach	23.54 (3.155)	0.435	<0.001	0.025	0.800	0.035	0.724	−0.002	0.984	−0.044	0.661
BACQ: Avoidance	15.49 (3.581)	−0.466	<0.001	−0.130	0.192	−0.179	0.071	−0.229	0.021	0.014	0.891

**Table 3:** Multiple linear regression models on Psychosocial Index.

Predictors	Psychosocial Index (*N =* 98)		
	*β*	95% *CI*	
ADL	0.145	−0.007	0.106
BACQ Approach	0.200 *	0.007	0.103
BACQ Avoid	−0.298 ***	−0.111	−0.030
Age			
60–64 (ref)			
65–69	0.240 *	0.104	0.867
70–74	0.223 *	0.088	0.846
75 and over	0.193 *	0.004	0.820
Gender			
Woman (ref)			
Man	−0.042	−0.403	0.231
Income			
Low (ref)			
Average	−0.252 *	−0.785	−0.078
High	0.047	−0.289	0.480
Health status			
Poor (ref)			
Medium to good	0.414 **	0.215	1.224
Excellent	0.509 **	0.372	1.488
COVID Contact			
No (ref)			
Yes	0.153	−0.056	1.250
COVID Symptom			
No (ref)			
At least one symptom	−0.049	−0.404	0.228

CI = Confidence Interval, (ref) = (reference). Only variables that were found to be significant in the correlation and univariate ANOVA analysis included in the regression model (see Table 1 and Table 2). * p < 0.05, ** *p* < 0.01, *** *p* < 0.001.

**Table 4:** Multiple linear regression models on cognitive outcomes.

Predictors	GNG No-go Accuracy *N* = 99			GNG Go Accuracy *N* = 101			LCT Accuracy *N =* 99			Cognitive Speed Index *N* = 99		
	*β*	95% *CI*		*β*	95% *CI*		*β*	95% *CI*		*β*	95% *CI*	
Psychosocial Index	0.144	−0.013	0.057	0.059	−0.008	0.013	0.022	−0.019	0.022	−0.132	−0.373	0.109
ADL	0.143	−0.003	0.018	−0.074	−0.004	0.002	−0.166	−0.011	0.001	−0.008	−0.072	0.066
BACQ Approach	−0.057	−0.011	0.006	−0.027	−0.003	0.002	−0.019	−0.005	0.005	−0.037	−0.068	0.048
BACQ Avoid	−0.100	−0.011	0.004	−0.106	−0.003	0.001	−0.228 *	−0.009	0.000	−0.029	−0.058	0.044
Age												
60–64 (ref)				X	X	X						
65–69	−0.137	−0.107	0.023	X	X	X	0.023	−0.033	0.041	0.087	−0.259	0.609
70–74	−0.113	−0.103	0.031	X	X	X	0.161	−0.014	0.070	0.185	−0.062	0.833
75 and over	−0.445 ***	−0.211	−0.075	X	X	X	−0.155	−0.068	0.011	0.435 ***	0.484	1.402
Gender												
Woman (ref)	X	X	X				X	X	X	X	X	X
Man	X	X	X	−0.156	−0.031	0.004	X	X	X	X	X	X
Education												
High school or lower (ref)	X	X	X				X	X	X	X	X	X
Post-secondary	X	X	X	0.115	−0.012	0.029	X	X	X	X	X	X
Graduate school	X	X	X	0.285	−0.001	0.045	X	X	X	X	X	X
Religion												
Christian/Catholicism (ref)	X	X	X	X	X	X				X	X	X
None	X	X	X	X	X	X	−0.021	−0.037	0.030	X	X	X
Other	X	X	X	X	X	X	−0.231 *	−0.081	−0.002	X	X	X
COVID Contact												
No (ref)				X	X	X	X	X	X	X	X	X
Yes	0.176	−0.045	0.253	X	X	X	X	X	X	X	X	X
COVID Travel												
No (ref)				X	X	X	X	X	X	X	X	X
Yes	−0.246	−0.325	0.001	X	X	X	X	X	X	X	X	X

CI = Confidence Interval, (ref) = (reference). “X” refers to the variables not included in the regression model for each psychosocial outcome based on the correlation and univariate ANOVA analysis (see Table 1 and Table 2). * *p* < 0.05, *** *p* < 0.001.

## Data Availability

Data are contained within the article.

## Abbreviations

The following abbreviations are used in this manuscript:

K10	Kessler-10
SWLS	Satisfaction with Life Scale
UCLA	UCLA Loneliness Scale Revised
GNG	Go/No-go Task
LCT	Letter Comparison Task
BACQ	Brief Approach/Avoidance Coping Questionnaire
ADL	Engagement in Activities of Daily Living
RT	Reaction Time
SES	Socioeconomic Status
MCI	Mild cognitive impairment
TIA	Transient ischemic attack
SBT	Short blessed test
ROC	Receiver Operating Characteristics
GAF	Global Assessment of Functioning
